# Self-Rated Health Status of Upper Secondary School Pupils and Its Associations with Multiple Health-Related Factors

**DOI:** 10.3390/ijerph19116947

**Published:** 2022-06-06

**Authors:** Armando Cocca, Martin Niedermeier, Vera Prünster, Katharina Wirnitzer, Clemens Drenowatz, Klaus Greier, Karin Labek, Gerhard Ruedl

**Affiliations:** 1 Department of Sport Science, University of Innsbruck, 6020 Innsbruck, Austria; martin.niedermeier@uibk.ac.at (M.N.); vera.pruenster@student.uibk.ac.at (V.P.); katharina.wirnitzer@ph-tirol.ac.at (K.W.); nikolaus.greier@uibk.ac.at (K.G.); gerhard.ruedl@uibk.ac.at (G.R.); 2 Department of Research and Development in Teacher Education, University College of Teacher Education Tyrol, 6010 Innsbruck, Austria; 3 Research Center Medical Humanities, Leopold-Franzens University of Innsbruck, 6020 Innsbruck, Austria; 4 Division of Sport, Physical Activity and Health, University of Education Upper Austria, 4020 Linz, Austria; clemens.drenowatz@ph-ooe.at; 5 Divison of Physical Education, Private Educational College (KPH-ES), 6422 Stams, Austria; 6 Institute of Psychology, University of Innsbruck, 6020 Innsbruck, Austria; karin.labek@uibk.ac.at

**Keywords:** youth, perceived health, physical fitness, structured sport activities, screen time

## Abstract

Health is an essential part of any individual, and gains particular importance in youth, as a good health at this age is more likely to reduce health risks both in the short and long term. The aim of this study was to assess the impact of physical and contextual parameters on youths’ perceived health. A total of 919 adolescents completed questionnaires on self-rated health status, electronic media use, leisure time and club physical activity, alcohol and tobacco consumption, and back pain, as well as performed the German Motor Performance Test. Participants with very good health had significantly higher physical fitness, leisure time exercise, and participated in sports clubs more often than those with poorer health. Electronic media use was significantly higher for those with poor/very poor health. Future intervention programs to improve youth health status should not only focus on active lifestyle but might also consider the impact of socioenvironmental factors, such as daily media use.

## 1. Introduction

Being healthy is an essential part of any adolescent life. In fact, youths with a positive health status have higher chances to grow into healthy adults, as several serious diseases (e.g., cardiorespiratory fitness, mental disorders) that generally occur in adulthood originate from health issues during adolescence [1]. Although several approaches exist to study people’s health status, individual self-perception has grown as one of the most interesting methods due to several advantages. Compared to medical records, self-perception tools are easier to apply, allow for encompassing broader strata of the population at lower costs, and have been shown to be a valid assessment strategy [2]. Although there exist some differences due to sociodemographic factors compared to the objective assessment of health, these two methods are deemed to obtain comparable findings [2].

Perceived health status is influenced by several socioenvironmental and personal factors. Studies found a positive association between physical fitness and perceived health in youth, especially in terms of cardiorespiratory and muscular fitness [3,4]. Participation in organized sports activities may also be an essential contributor to perceived health as there exists a positive relationship between sports participation and perceived health regardless of the sociocultural environment [5,6]. In addition, children with healthier body mass index (BMI) display enhanced perceived health status or higher perception of health-related self-efficacy [7,8]. Furthermore, BMI and weight status may indirectly affect perceived health through associations with active habits and nutritional and lifestyle choices [9].

On the other hand, unhealthy behaviors, such as tobacco and alcohol consumption, excessive screen time and electronic media use, and back pain, can be mentioned as prominent sources of reduced health perception in youth [9,10,11]. Tebar et al. [12] showed a worse health perception in youth with a higher number of sedentary activities, regardless of their physical activity habits. Prolonged sitting and excess screen time are also related to back pain at early ages [13,14], which has a detrimental effect on perceived health as well [11,15].

Although previous studies have investigated the separate effect of the factors mentioned earlier on perceived health, to date, there are only a few studies addressing their intertwined action. Perceived health, however, is the result of the combination of physical, social, environmental, and demographic agents, and its alteration cannot be fully understood without considering this wide spectrum of variables. The aims of this study, therefore, are to evaluate the current perceived health of upper secondary school pupils and its associations with multiple health-related factors; to compare physical, social, and environmental variables by health status; and to analyze the explanatory power of such variables on perceived health status.

## 2. Materials and Methods

### 2.1. Participants

Using a cross-sectional design, adolescents between grades 9 and 12 from six randomly selected public secondary schools participated in the study. Data collection occurred during spring 2018. The study protocol received formal ethical approval. Parents provided written informed consent, and participants provided assent at the time of data collection. All study procedures were in accordance with the ethical standards of the Declaration of Helsinki.

### 2.2. Anthropometric Measurements

In line with previous studies on Austrian adolescents [16,17], students’ height and weight were measured in sport clothing and barefoot during regular physical education lessons in the school gym. Body height was measured with a mobile stadiometer “Seca 217” (Seca, Hamburg, Germany) with an accuracy of 0.1 cm, and body weight was measured with a calibrated scale “Grundig PS 2010” (Grundig AG, Neu-Isenburg, Germany) with an accuracy of 0.1 kg. BMI (kg/m^2^) was calculated, and students were classified according to the BMI reference system by Kromeyer-Hauschild et al. [18] into three groups: underweight vs. normal weight vs. overweight/obese.

### 2.3. Questionnaire

Regarding the perceived health status of adolescents, self-rated health represents a meaningful subjective indicator for general health [19]. According to the Health Behavior in School-Aged Children (HBSC) study [20], self-rated health was measured by a single item with the question “Would you say your health in general is …”. For answering this question, we used a five-point Likert scale (very good, good, moderate, poor, very poor). Participants were subsequently divided into four health categories (very good, good, moderate, poor/very poor), which are comparable to the four HBSC categories of self-rated health (excellent, good, fair, poor) [20]. Participants also reported their average daily time (hours/day) of electronic media use (smartphone, tablet, computer, TV, etc.) outside of school as well as the presence of a TV in their bedroom [16]. Sports club participation was determined via self-report (yes/no). In addition, participants reported mean duration (hours per week) of practicing sports in a club setting as well as during leisure time. Weekly hours of sports club participation and weekly hours of physical activity in leisure time were then summed up to obtain a total amount of weekly leisure time sports activity. Finally, smoking and alcohol consumption, as well as current back pain during the previous 7 days, were reported using a dichotomous variable (yes/no), respectively.

### 2.4. Physical Fitness

For testing the physical fitness, participants completed the German Motor Performance Test (GMT) 6-18 [21]. The GMT is a standardized test battery consisting of eight items that assesses various subdomains of physical fitness: 20 m sprint (sprint velocity), balancing backwards on three 3 m long beams with different width (coordination in a task requiring precision), jumping sidewards over a middle line for 15 s (coordination under time pressure), stand-and-reach (flexibility), push-ups in a period of 40 s (strength endurance), sit-ups in a period of 40 s (strength endurance), standing long jump (power), and 6 min run (endurance). With regard to the performance criteria of GMT 6-18, the inter-rater reliability (0.95) and test–retest reliability (0.82) of the test battery were good, and the battery has been validated for assessing speed, coordination, flexibility, strength, and endurance [21]. According to the exact instruction of the test manual by Bös [21], tests were carried out by trained physical education students in the gymnasiums of the participating schools. All tests were completed during a single session, lasting about 90 min in random order, except for the 20 m sprint, which was completed at the beginning, and the 6 min run, which was completed at the end of the testing session. Values of the eight test items were standardized according to age and sex-reference values resulting in so-called Z-values, with a value of 100 representing average performance in the tests. According to Bös [21], the formula for the standardization is

Z = (x_i_ − M)/SD × 10 + 100 (1)

where x_i_ is the raw value of the test item, M is the mean, and SD the standard deviation of the age- and sex-specific norm sample. Values above 100 indicate above-average performance and values below 100 indicate below-average performance. The average of all scores was used as an indicator for overall physical fitness [21].

### 2.5. Statistics

Data are presented as means ± standard deviations and relative (absolute) frequencies, respectively. All statistical analyses were conducted using SPSS Statistics version 26 (IBM, New York, NY, USA). The first step of the analysis consisted of tests on differences in potentially associated factors between the four health groups group with very good perceived health (VH), good perceived health (GH), moderate perceived health (MH), and poor/very poor perceived health (PH). Since the group sizes were unequal (n = 34 for the smallest and n = 482 for the largest group), Kruskal–Wallis H-tests were calculated for continuous factors (age, BMI, mean daily electronic media use, mean weekly leisure time sports activity, and mean physical fitness). For categorical factors (sex, weight status, electronic media in bedroom, sports club participation, and prevalence of smoking and drinking alcohol, as well as of suffering from back pain), Pearson’s chi-square tests were used to evaluate differences between the four health groups. In the case of a significant result, additional post hoc tests with Bonferroni correction were performed.

The second analysis step consisted of a multiple multinominal logistic regression analysis with health status as the dependent variable. A multinominal logistic regression model was chosen since proportional odds between categories of health status were not assumed. The reference level was set to the group with very good perceived health (VH). All variables with significant differences between categories were included as predictor variables to the multiple model, except for weight status to avoid redundancy with BMI. Although not significant in the simple analysis, age was included in the multiple model to account for age differences in the predictor variables (e.g., percentage of smokers, percentage of alcohol consumers, BMI). Odds ratios (OR), including 95% confidence intervals (95% CI), were calculated for all predictor variables. 

All *p*-values were two-tailed, and values less than 0.05 were considered to indicate statistical significance.

## 3. Results

A total of 919 adolescents (55.6% girls) with a mean age of 15.5 ± 1.3 years and a mean BMI of 22.0 ± 3.9 kg/m^2^ participated. With regard to the additional classification into three weight groups, 5.3% (n = 49) of students were in the underweight group, 75.3% (n = 692) in the normal weight group, and 19.4% (n = 178) in the overweight/obese group, respectively. 

Regarding self-rated health status, 21.8% of adolescents stated very good health, 52.6% good, 21.9% moderate, and 3.7% poor/very poor health.

In total, 68% stated to have a TV or computer in the bedroom, and 42% participated in sports clubs. Additionally, 8.7% reported smoking, 59.8% consumed alcohol, and 42.2% reported suffering from back pain.

Mean reported daily electronic media use was 2.7 ± 1.7 h, mean reported weekly leisure time sports activity was 9.8 ± 4.9 h, and mean physical fitness (Z-value) of the cohort was 105.6 ± 6.2.

In Table 1, results of the univariate comparison of the four different health groups are presented. Significant differences between groups were found with regard to sex, BMI, weight status, electronic media in the bedroom, daily electronic media use, sports club participation, weekly leisure time sports activity, physical fitness, smoking, and prevalence of back pain.

In Table 2, results of the multiple multinomial regression model are presented. Compared to the group with very good perceived health (reference group), being female (OR = 1.57) significantly increased the odds for being in the group with good perceived health. Conversely, being older (OR = 0.84), having electronic media in the bedroom (OR = 0.57), showing higher physical fitness (OR = 0.93), and not reporting back pain (OR = 0.66) significantly decreased the odds for being in the group with good perceived health compared to the group with very good perceived health. 

Compared to the group with very good perceived health (reference group), the likelihood for being in the group with moderate perceived health significantly increased with higher BMI (OR = 1.11) and smoking (OR = 3.17). Conversely, being older (OR = 0.81), participating in a sports club (OR = 0.41), higher weekly leisure time sports activity (OR = 0.89), higher physical fitness (OR = 0.88), and not reporting back pain (OR = 0.44) was associated with a decreased likelihood to be in the group with moderate perceived health.

Similarly, the odds for being in the poor/very poor health group was significantly larger in adolescents with higher BMI (OR = 1.22) compared to the group with very good perceived health. Age (OR = 0.70), sports club participation (OR = 0.23), higher weekly leisure time sports activity (OR = 0.88), higher physical fitness (OR = 0.86), and not reporting back pain (OR = 0.20), on the other hand, was associated with decreased odds for being in the group with poor/very poor health perceived health compared to the group with very good perceived health. Additionally, the chance for an increased daily electronic media use was significantly higher in the poor/very poor health group (OR = 1.30).

## 4. Discussion

The aims of this study were to evaluate the current perceived health of upper secondary school students and its associations with multiple health-related factors; to compare physical, social, and environmental variables by health status; and to analyze the explanatory power of such variables on perceived health status. 

Our findings show that almost three quarters of the studied population (74.4%) perceive their health as at least good. In accordance, the latest Austrian Health Interview Survey [22] reported that the vast majority of adolescents reported being satisfied with their health status, with less than 10% considering their health as poor or very poor.

A more detailed analysis of the results indicates that a high perception of health (very good, good) is very similar in male and female participants (44.5% and 57.9% of girls, respectively); however, two thirds of the “moderate health” group is represented by girls. This seems to be partially in line with previous studies suggesting that women tend to show a poorer perception of their health [23,24] or similar perception to men, despite the latter reporting higher incidence of health issues [25].

When analyzing the factors associated with perceived health in youth, the positive association between physical fitness and perceived health is clear. In fact, physical fitness seems to have a significant influence at all levels of health, as indicated by various studies [3,4]. In line with our findings, Liu et al. [26] proposed a significant association between overall physical fitness and general health, which, together, may predict adolescents’ lifestyle choices and their willingness to engage in and promote healthy habits. The prominent role of physical fitness is also emphasized in studies focusing on different areas of youth’s individual health, such as mental health [27], physical and metabolic health [28], social health [29], and overall quality of life [4]. Physical fitness, therefore, should be promoted in different settings, including the school environment, physical education, and leisure time. 

Accordingly, leisure time physical activity appears to be an important determinant for perceived health, particularly discriminating between positive and low or negative perception of health [30]. Gomes et al. [31] report that leisure time exercise may positively influence mental health both directly and indirectly by reducing the time spent by youth in sedentary activities. These types of activities are also bidirectionally associated with health literacy, which may not only improve adolescents’ health status, but also their knowledge and understanding of the indicators of health and, as a consequence, influence their lifestyle choices [32].

Our sample reported significantly better health for those engaging in sports club activities compared to those who did not. Participation in sports clubs, in fact, may be important not only for individual health but also as an opportunity for youth to reduce social and environmental inequities [33].

Similar to our findings on leisure time and organized physical activity, in our study, worse BMI scores were only associated with lower to poor perception of health, with no significant differences between those participants who reported being in very good or good health. This is in line with previous studies showing the impact of BMI and weight status on perceived health [7,9]. Additionally, BMI is considered a mediator of the relation between participation in physical activity and perceived health [9].

It may have been surprising that having electronic media in the bedroom did not affect the perception of health in our sample. Although some studies suggest a relation between media availability in the bedroom and certain factors associated with general health, such as sleep time and quality [34], increased screen time [35], or even depression symptoms [36], the availability of electronic media in the bedroom does not necessarily lead to their usage or over-usage. In our sample, the fact that most of the participants stated to have media available in their bedroom regardless of the health group they belonged to might simply indicate that they do not make excessive use of them. However, when we combine these results with those on the daily usage of media, the difference between adolescents in the “very good health” group and those in the “moderate” and “poor health” ones becomes significant. It is also evident from our data that only the “very good health” group members (and partially the “good health” group ones) are close to the recommendations on maximum daily screen time (2 h/day) [37], whereas those who perceived lower or poor health spent an average of 3 h and 4 h on screen, respectively. Excessive screen time, therefore, should be considered as correlation of the perception of one’s own health, as already stated elsewhere [10,12].

In addition, reported back pain is significantly lower in the “very good health” group compared to all the other groups. This is in line with previous studies on the impact of this issue on perceived health [11,15]. As the authors emphasize, back pain is logically strictly related to people’s consideration of their own health as positive or negative. In fact, differently than physical fitness, physical activity, and body composition, which may be perceived very differently based on cultural aspects [38], and screen time, which some individuals may at times perceive as positive for their mental health [39], back pain is embedded in one’s health condition, and may be even considered as a component of it. Therefore, it is reasonable to assume that those who did not report any back pain consider their health better compared to those who experienced such a problem. Finally, it is important to keep in mind that our results on back pain may not be sufficiently accurate, as the onset and duration of this issue (for instance, chronic vs. acute), as well as its intensity and source (mechanical, neurological, etc.), were not considered in the present study.

### Limitations

This study presents some limitations. Although perceived health status evaluated with a single item is considered a valid measurement method, using self-rating tools may encompass risks, such as invalid responses, social desirability biases, or general response biases [40]. Additionally, health status has been evaluated with a single item. Given the complex structure of such a variable, a single item might not reflect such condition in full. However, we decided not to burden the participants with an excessively long series of items, in accordance with previous literature on the topic [41,42], and based it on single-item assessment that has been validated [43]. Additionally, our analysis of potential agents of perceived health could not include several other variables that are also known to be associated with it, such as personality traits [44], dietary patterns [45], or family situation and social support networks [46].

In the future, this type of study could also expand to explore potential differences in such network of variables based on school location (for instance, type of neighborhood, urban vs. rural) or school type (public or private). Another interesting addition to this research could be represented by a deeper analysis of participants’ sports habits both within sports clubs and during leisure time, since the type of exercise is known to potentially affect health-related parameters [47].

## 5. Conclusions

Perceived health status may be influenced by many factors, both environmental and personal. Among them, the overall level of physical fitness, which includes cardiorespiratory endurance, muscular strength, and flexibility, among others, seem to be central determinants for the perception of adolescents’ health. Accordingly, sports club participation and overall leisure time physical activities appear to be critical correlates of perceived health. In addition, screen time needs to be considered in strategies targeting health in adolescents. Therefore, future investigations/approaches should be multifocal and include robust physical fitness developmental plans through physical education curricula in school while also emphasizing participation in organized sport club activities and replacing sedentary leisure time, such as screen time, with more active tasks. All these attempts/interventions may enhance youth health literacy as well, which could reduce other detrimental health behaviors such as smoking and alcohol consumption. Any intervention should also include parental counseling and a coordinated effort of families, teachers, and administrators in order to pursue appropriate strategies targeting the diverse contributors of adolescents’ health.

## Figures and Tables

**Table 1:** Group differences in factors between adolescents with a very good, good, moderate, and poor/very poor perceived health. Values are means with SD or prevalence (%).

Factors	Very Good Health (n = 200)	Good Health (n = 482)	Moderate Health (n = 201)	Poor/Very Poor Health (n = 34)	*p*-Value	Post Hoc ^c^
Age [years]	15.6 ± 1.3	15.5 ± 1.3	15.5 ± 1.3	15.4 ± 1.6	0.691 ^a^	none
Sex [%], girls	44.5	57.9	61.7	50.0	0.002 ^b^	VH:GH, VH:MH,
BMI [kg/m^2^]	21.3 ± 2.7	21.6 ± 3.4	22.8 ± 4.5	25.9 ± 7.1	<0.001 ^a^	VH:MH, VH:PH, GH:MH, GH:PH
Weight status [%]					<0.001 ^b^	VH:MH, GH:MH
Underweight	3.5	5.8	7.0	0		
Normal weight	87.0	77.2	63.2	52.9		
Overweight/adipose	9.5	17.0	29.9	47.1		
Electronic media in bedroom [%], yes	72.5	63.1	74.1	76.5	0.008 ^b^	GH:MH
Daily electronic media use [h]	2.4 ± 1.3	2.6 ± 1.7	2.9 ± 1.7	3.8 ± 2.7	<0.001 ^a^	VH:MH, VH:PH
Sports club participation [%], yes	59.0	43.4	25.4	23.5	<0.001 ^b^	VH:GH, VH:MH, VH:PH, GH:MH
Weekly leisure time sports activity [h]	8.0 ± 5.4	6.2 ± 5.0	3.9 ± 3.7	3.1 ± 4.1	<0.001 ^a^	VH:GH, VH:MH, VH:PH, GH:MH, GH:PH
Physical fitness [Z-value]	108.4 ± 5.4	106.0 ± 5.8	102.9 ± 5.6	99.7 ± 9.1	<0.001 ^a^	VH:GH, VH:MH, VH:PH, GH:MH, GH:PH
Smoking [%], yes	4.0	8.5	13.9	5.9	0.005 ^b^	VH:MH
Alcohol consumption [%], yes	56.5	61.2	61.2	52.9	0.546 ^b^	none
Back pain [%], yes	35.0	41.3	47.8	61.8	0.006 ^b^	VH:PH

Notes: Data are displayed as means ± standard deviations or relative frequencies, as appropriate. ^a^: Kruskal–Wallis H-test, ^b^: chi-square-test, ^c^: significant differences specified according to Bonferroni-corrected pairwise comparisons. BMI: body mass index, VH: group with very good perceived health, GH: group with good perceived health, MH: group with moderate perceived health, PH: group with poor/very poor perceived health. Bold values indicate significant differences.

**Table 2:** Results of the multiple multinomial regression analysis with the dependent variable perceived health status.

Variable	B	SE B	OR	OR 95% CI lb	OR 95% CI ub	*p*
**GH vs. VH**						
Intercept	11.52	(2.54)				<0.001
Age [years]	−0.17	(0.07)	0.84	0.74	0.97	0.015
Sex, female	0.45	(0.19)	1.57	1.09	2.27	0.016
Body mass index [kg/m^2^]	0.04	(0.03)	1.04	0.98	1.11	0.170
Electronic media in bedroom, yes	−0.56	(0.20)	0.57	0.39	0.84	0.004
Daily electronic media use [h]	0.10	(0.06)	1.10	0.97	1.25	0.129
Sports club participation, yes	−0.61	(0.32)	0.54	0.29	1.02	0.057
Weekly leisure time sports activity [h]	−0.03	(0.02)	0.97	0.94	1.01	0.166
Physical fitness [Z-value]	−0.08	(0.02)	0.93	0.90	0.96	<0.001
Smoking, yes	0.73	(0.41)	2.08	0.94	4.63	0.072
Back pain, no	−0.42	(0.19)	0.66	0.46	0.95	0.024
**MH vs. VH**						
Intercept	15.79	(3.12)				<0.001
Age [years]	−0.21	(0.08)	0.81	0.69	0.96	0.014
Sex, female	0.42	(0.24)	1.52	0.96	2.42	0.074
Body mass index [kg/m^2^]	0.10	(0.03)	1.11	1.03	1.18	0.003
Electronic media in bedroom, yes	−0.24	(0.25)	0.79	0.48	1.30	0.351
Daily electronic media use [h]	0.11	(0.07)	1.12	0.97	1.30	0.124
Sports club participation, yes	−0.89	(0.36)	0.41	0.20	0.83	0.012
Weekly leisure time sports activity [h]	−0.12	(0.03)	0.89	0.84	0.94	<0.001
Physical fitness [Z-value]	−0.13	(0.02)	0.88	0.84	0.92	<0.001
Smoking, yes	1.16	(0.44)	3.17	1.33	7.55	0.009
Back pain, no	−0.83	(0.23)	0.44	0.28	0.68	<0.001
**PH vs. VH**						
Intercept	17.48	(5.49)				0.001
Age [years]	−0.36	(0.16)	0.70	0.51	0.95	0.022
Sex, female	−0.06	(0.44)	0.94	0.40	2.21	0.883
Body mass index [kg/m^2^]	0.20	(0.05)	1.22	1.11	1.34	<0.001
Electronic media in bedroom, yes	−0.59	(0.49)	0.55	0.21	1.46	0.233
Daily electronic media use [h]	0.26	(0.10)	1.30	1.06	1.59	0.013
Sports club participation, yes	−1.48	(0.56)	0.23	0.08	0.68	0.008
Weekly leisure time sports activity [h]	−0.13	(0.07)	0.88	0.77	1.00	0.047
Physical fitness [Z-value]	−0.15	(0.04)	0.86	0.80	0.93	<0.001
Smoking, yes	0.19	(0.86)	1.20	0.22	6.49	0.829
Back pain, no	−1.61	(0.43)	0.20	0.09	0.46	<0.001

Notes: R^2^ (Nagelkerke) = 0.26, model chi-square (21) = 243.45, *p* < 0.001, B: unstandardized regression coefficient, SE: standard error, OR: odds ratio, 95% CI: 95% confidence interval, lb: lower bound, ub: upper bound. Bold values indicate *p* < 0.05. VH: group with very good perceived health (reference group), GH: group with good perceived health, MH: group with moderate perceived health, PH: group with poor/very poor perceived health.

## Data Availability

Data from this study are not public due to privacy policies and are available upon request to the corresponding author.
